# Autoantibodies directed against α1-adrenergic receptor and endothelin receptor A in patients with prostate cancer

**DOI:** 10.1186/s13317-020-00136-y

**Published:** 2020-09-25

**Authors:** Gerd Wallukat, Burkhard Jandrig, Niels-Peter Becker, Johann J. Wendler, Peter Göttel, Johannes Müller, Martin Schostak, Ingolf Schimke

**Affiliations:** 1 Berlin Cures GmbH, Knesebeckstraße 59-61, 10719 Berlin, Germany; 2 Universitätsklinik für Urologie, Uroonkologie, robotergestützte und fokale Therapie, Otto von Guericke Universität, Magdeburg, Germany

**Keywords:** Aptamer, Anti-α1-adrenergic receptor autoantibodies, Anti-endothelin receptor A autoantibodies, Functional autoantibodies, G-protein coupled receptor, Prostate cancer

## Abstract

**Background:**

For prostate cancer, signaling pathways induced by over-boarding stimulation of G-protein coupled receptors (GPCR) such as the endothelin, α1- and β-adrenergic, muscarinic and angiotensin 1 receptors were accused to support the carcinogenesis. However, excessive receptor stimulation by physiological receptor ligands is minimized by a control system that induces receptor sensitization and down-regulation. This system is missing when so-called “functional autoantibodies” bind to the GPCR (GPCR-AAB). If GPCR-AAB were found in patients with prostate cancer, uncontrolled GPCR stimulation could make these autoantibodies an additional supporter in prostate cancer.

**Methods:**

Using the bioassay of spontaneously beating cultured rat neonatal cardiomyocytes, GPCR-AAB were identified, quantified and characterized in the serum of 25 patients (aged 56–78 years, median 70 years) with prostate cancer compared to 10 male patients (aged 48–82 years, median 64) with urinary stone disorders (controls).

**Results:**

Of the cancer patients, 24 (96%) and 17 (68%), respectively, carried autoantibodies directed against the α1-adrenergic receptor (α1-AAB) and endothelin receptor A (ETA-AAB). No patient was negative for both GPCR-AAB. In contrast, ETA-AAB and α1-AAB were absent in all (100%) and 9 (90%) of the 10 control patients, respectively. While α1-AAB targeted a specific epitope of the first extracellular loop of the α1-adrenergic receptor subtype A, an epitope of the second extracellular loop of the ETA receptor was identified as a target of ETA-AAB. As demonstrated in vitro, the functional activity of both autoantibodies found in prostate cancer can be neutralized by the aptamer BC007.

**Conclusions:**

We hypothesized that α1-AAB and ETA-AAB, which are highly present in prostate cancer patients, could by their functional activity support carcinogenesis by excessive receptor stimulation. The in vitro demonstrated neutralization of α1- and ETA-AAB by the aptamer BC007 could open the door to complement the treatments already available for prostate cancer.

## Introduction

For prostate cancer, one of the most common cancers in men [1] various pathogenic players are discussed, including signaling pathways mediated by G-protein coupled receptors (GPCR). Among the signaling pathways, those linked to endothelin [2–5], α1-adrenergic [6, 7], β-adrenergic [7, 8], muscarinic [9–11], and angiotensin 1 receptors [12, 13], have been considered in relation to cell proliferation, metastasis and angiogenesis. As a key to explaining the potency of GPCR-mediated signaling in carcinogenesis, hyper-stimulation by its physiological receptor ligands is accused.

To prevent or minimize hyper-stimulation of the GPCR-mediated signaling by their physiological ligands, cells have developed a complex system of mechanisms including GPCR desensitization and down-regulation with a pivotal role of GPCR kinases and β-arrestin as summarized in [14]. Unfortunately, this GPCR self-controlling system does not work if so-called “functional autoantibodies” (GPCR-AAB) bind to the GPCR. This is well-documented for autoantibodies directed against the β1-adrenergic receptor and the muscarinic 2 receptor, but has been more and more accepted as a general phenomenon of GPCR-AAB as summarized in [15].

Consequently, GPCR-AAB cause uncontrolled and over-boarding stimulation of the signaling pathways. This leads to the long-time perpetuation of downstream effects, making the GPCR-AAB a potent pathogenic driver or supporter [16, 17]. Among the diseases which are thought to be related to GPCR-AAB-associated effects, cardiovascular diseases [18] are predominant but not exclusive, as exemplarily demonstrated for patients with dementia [19].

Recently we published that patients with benign prostatic hyperplasia/low urinary tract syndrome (BPH/LUTS) carry GPCR-AAB, especially those directed against the endothelin receptor A (ETA-AAB), and have discussed this finding in the context of vascular pathophysiology and cell growth [20]. In the present study we took a look at the GPCR-AAB situation in patients with prostate cancer where we found ETA-AAB as for patients with BPH/LUTS, but additionally GPCR-AAB directed against the α1-adrenergic receptor (α1-AAB).

In addition, we characterized the GPCR-AAB in relation to the target region on their GPCR and showed in an in vitro experiment that the functional activity of GPCR-AAB can be neutralized, which opens a way to counteract GPCR-AAB in patients with prostate cancer.

## Materials and methods

### Patients

Of 25 patients (age 56–78 years, median 70 years) with prostate cancer (diagnosed based on [21]) (study group) and 10 male patients (age 48–82 years, median 64 years) with nephrolithiasis or urolithiasis (control group) treated at the Universitätsklinik für Urologie, Uroonkologie, robotergestützte und fokale Therapie, Otto von Guericke Universität Magdeburg, Germany, for prostate resection or lithotripsy/endoscopic removal, the serum collected for the certified bio-banking was analyzed. All patients signed informed consent forms approved by the Medical Ethics Committee of the Otto-von-Guericke University Magdeburg (# 87/11). Clinic-pathological data of prostate cancer patients and controls can be seen in Table 1.

**Table 1:** Clinic-pathological data of prostate cancer patients and controls

	PCa	Control
Number individuals	25	10
Mean/median age (years)	68.9/69.9 (56.3–78.2)	65.0/64.1 (47.8–81.8)
Mean/median height (cm)	179/178 (168–196)	177/179 (165–190)
Mean/median weight (kg)	87/84 (64–110)	94/90 (80–117)
Mean/median BMI	27.1/26.2 (21.5–33.1)	30.0/29.9 (23.0–36.1)
Mean/median PSA (ng/ml)	238.1/21.5 (6.2–3487)	
Gleason score		
3 + 5	2	
4 + 4	13	
4 + 5	5	
5 + 4	2	
5 + 5	3	
Gleason sum		
8	15	
9	7	
10	3	
Grade groups (ISUP)		
4	15	
5	10	
Grading (c/p)		
T2a	1	
T2b	1	
T2c	5	
T3	4	
T3a	4	
T3b	10	
Lymph node metastasis pN0/pN1	9/6	
Distant metastasis pM0/pM1	11/4	

### Analytics

#### Measurement of GPCR-AAB activity

To identify and quantify the GPCR-AAB, localize its receptor binding site and demonstrate the ability to neutralize the functional activity of the GPCR-AAB activity, the bioassay of spontaneously beating cultured neonatal rat cardiomyocytes was used where the cells’ chronotropic response to patients’ IgG containing the GPCR-AAB was recorded (for the mechanistic background of the assay, its structure, standardization and necessary sample preparation see [22, 23]). As defined: 1 beat/min frequency increase = 1 unit GPCR-AAB activity (positive chronotropy) and 1 beat/min frequency decrease = − 1 unit GPCR-AAB activity (negative chronotropy). The lower limits of detection (LLD) for positive and negative chronotropic GPCR-AAB were calculated as 4.0 U and − 4.0 U, respectively. Based on x ± 3 SD of the GPCR-AAB level of more than 100 healthy volunteers, cut offs at ≥ 8.0 U and ≤ − 8.0 U were defined for patient who are positive for positive chronotropic GPCR-AAB and negative chronotropic GPCR-AAB, respectively.

**Fig. 1:**
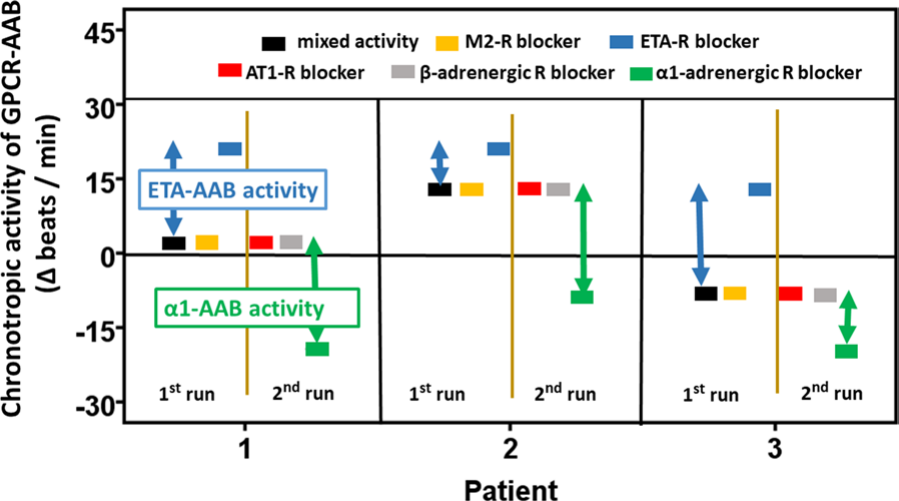
Measurement strategy for autoantibodies directed against G-protein coupled receptors (GPCR-AAB) in the IgG of patients with prostate cancer using the bioassay of cultured spontaneously beating neonatal rat cardiomyocytes. The bioassay monitored the chronotropic activity of IgG resulting from the presence of positive and negative chronotropic GPCR-AABs. For GPCR-AAB differentiation and activity calculation, the bioassay was performed in two runs. The first run was performed in the absence of GPCR blockers and thereafter with successive addition of 0.1 µmol/l atropine and 0.1 µmol/l BQ123 to block the muscarinic receptor 2 and endothelin receptor A. The second run was performed in the absence of GPCR blockers and thereafter with successive addition of 0.1 µmol/l losartan, 0.1 µmol/l propranolol and 0.1 µmol/l prazosin to block the AT1, β-adrenergic, and α1-adrenergic receptors. Calculation of the GPCR-AAB activity: mixed GPCR-AAB activity = M2-AAB activity + ETA-AAB activity + β-AAB activity + AT1-AAB activity + α1-AAB activity. Blue arrows indicate the ETA-AAB activity. Green arrows indicate the α1-AAB activity

Through the intelligent use of GPCR blockers (exemplarily illustrated in Fig. 1 for three patients) the chronotropic response of the patients’ IgG can be attributed to the respective GPCR-AAB. For the GPCR-AAB possibly relevant for prostate cancer as indicated in the introduction, the following blockers were used: 0.1 µmol/l BQ123 for ETA-AAB, 0.1 µmol/l atropine for M2-AAB, 0.1 µmol/l losartan for AT1-AAB, 0.1 µmol/l propranolol for β-AAB, and 0.1 µmol/l prazosin for α1-AAB. Consequently, the GPCR-AAB activity was calculated based on the equation “mixed GPCR-AAB activity = M2-AAB activity + ETA-AAB activity + β-AAB activity + AT1-AAB activity + α1-AAB activity”.

#### Localizing of the receptor binding site

To localize the extracellular binding site (extracellular loops) and map the specific epitope of the α1 adrenergic and ETA receptors targeted by α1-AAB and ETA-AAB, the bioassay was performed with IgG (GPCR-AAB) pre-incubated with an excess of peptides (1 µg peptide per 1 µg IgG protein) synthesized by Biosyntan GmbH, Berlin-Buch, Germany. As indicated in Table 2, blocking peptides were used representing the first, second and third extracellular loop of the ETA and α1-adrenergic receptor as well as peptides, which overlapped to represent the amino acid sequence of the first extracellular loop of the α1-adrenergic and the second extracellular loop of the ETA receptor [23].

**Table 2:** Peptides used to localize (loop analysis, epitope mapping) the binding site of the endothelin receptor A and the α1-adrenergic receptor targeted by ETA-AAB and α1-AAB

A	Endothelin receptor A	α1-adrenergic receptor
Loop 1	^134^LPINVFKLLAGRWPFDHNDFGVFLCKL^160^	^89^LGYWAFGRVFCN^100^
Loop 2	^229^FEYRGEQHKTCMLNATSKFMEFYQDVKD^256^	^168^PAPEDETICQINEE^181^
Loop 3	^329^KKTVYNEMDKNRCELLLSFLL^348^	^298^FPDFKPSETVFKIVIFWLGYLNSC^329^
B	Endothelin receptor A second loop	α1-adrenergic receptor first loop
Loop	FEYRGEQHKTCMLNATSKFMEFYQDVKD	LGYWAFGRVFCN
P1	FEYRGEQ	FWAFGR
P2	EQHKTCM	GRVFCDV
P3	MLNATSK	
P4	SKFMEFY	
P5	FYQDVKD	

A: Peptides representing the first, second and third extracellular loops of the endothelin receptor A and the α1-adrenergic receptor. B: Peptides with overlapping loop sequence representing the second extracellular loop (endothelin receptor A) and the first extracellular loop (α1-adrenergic receptor)

ETA and α1-adrenergic receptor (ETA receptor: ^134^LPINVFKLLAGRWPFDHNDFGVFLCKL^160^, ^229^FEYRGEQHKTCMLNATSKFMEFYQDVKD^256^, and ^329^KKTVYNEMDKNRCELLLSFLL^348^); (α1-adrenergic receptor: ^89^LGYWAFGRVFCN^100^, ^168^PAPEDETICQINEE^181^, and ^298^FPDFKPSETVFKIVIFWLGYLNSC^329^)

To demonstrate GPCR-AAB binding sites and the GPCR-AAB neutralization (see below), representative experiments were performed with a randomly selected sample size > 10% (samples of 4 and 5 patients, respectively) of the population size of 25 patients.

#### ETA-AAB and α1-AAB in vitro neutralization experiments

The bioassay was also used to demonstrate the neutralization of α1-AAB and ETA-AAB activity by the aptamer BC007 [24].

## Results

### Basics

The study group of 25 patients with prostate cancer (age/years: median = 70, min = 56, max = 78; Gleason score: median = 8, min = 8, max = 10) and the control group of 10 patients with urinary stone disorders (aged 48–82 years, median 64 years) were not significantly different in age. All patients with prostate cancer presented with a Gleason score ≥ 8.

### Analytics

Figure 1, exemplarily for three patients, illustrates the measurement procedure of the GPCR-AAB activity in prostate cancer patients by means of the bioassay of spontaneously beating cultured neonatal rat cardiomyocytes. Based on bioassay handling, described in Materials and methods, one would assume, that patient 1 (black line) was negative for GPCR-AAB (activity < LLD), the second patient had positive chronotropic IgG and the third patient had negative chronotropic IgG. However, all three patients may represent a mixed activity, since the IgG of the patients could carry both positive and negative chronotropic GPCR-AAB, which may partially (patient 2 and 3) or completely (patient 1) cancel each other out. Therefore, an in-depth analysis with successive blocking experiments with receptor antagonists was necessary to find out which GPCR-AAB contributes to mixed activity. As shown in Fig. 1, a first bioassay run was performed to look for negative chronotropic GPCR-AAB, such as autoantibodies directed against the muscarinic receptor 2 (M2-AAB) and ETA-AAB. Blockade of the muscarinic receptor 2 (yellow line) with atropine did not affect the beat rate of the cells as compared with the unblocked rate (mixed activity), which excluded the presence of M2-AAB. After successive blockade of the endothelin A receptor with BQ123 (blue line), in contrast, the cells responded to the IgG of patient 1 and patient 2 with increased beat rate. For patient 3, the beat rate switched from decreased to increased rate, respectively. This indicated the presence of ETA-AAB in the IgG of all three patients. To assign the positive chronotropic activity to a specific GPCR-AAB and to calculate this activity, the bioassay was performed in a second run with successive addition of blockers for AT1 (red line), β-adrenergic (grey line), and α1-adrenergic receptors (green line). AT1 and β-adrenergic receptor blockers losartan and propranolol, respectively, had no effect on the mixed activity of the patients’ IgG, indicating the absence of AT1-AAB, β1-AAB, and β2-AAB in the patients’ IgG. In contrast, blocking the α1-adrenergic receptor with prazosin reduced the beat rate of cells as an indicator of α1-AAB in the patients’ IgG.

### Autoantibody pattern

**Fig. 2:**
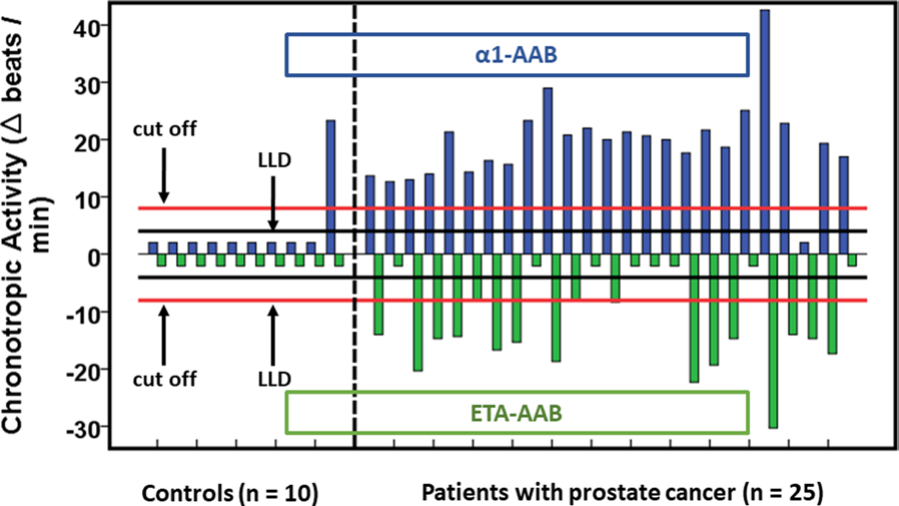
Chronotropic activity (∆ beats/min) of autoantibodies directed against the α1-adrenergic receptor (α1-AAB) and endothelin receptor A (ETA-AAB) in patients with prostate cancer as compared with control patients suffering from urinary stone disorders. For measurement, the bioassay of spontaneously beating cultured neonatal rat cardiomyocytes was performed in the presence of the patients’ IgG without and with the presence of specific receptor blockers (endothelin receptor A: 0.1 µmol/l BQ 123, muscarinic 2 receptor: 0.1 µmol/l atropine, β-adrenergic receptor: 0.1 µmol/l propranolol, AT-1 receptor: 0.1 µmol/l losartan, α1-adrenergic receptor: 0.1 µmol/l prazosin). Values below the lower limit of detection (LLD) were displayed as half range values. LLD = Δ 4 beats/min for α1-AAB, Δ − 4 beats/min for ETA-AAB; cut off (separating healthy from disease subjects) = Δ 8 beats per/min for α1-AAB, Δ − 8 beats/min for ETA-AAB

Of the 25 cancer patients (Fig. 2), 24 (96%) presented with a α1-AAB level above the cut off ≥ 8.0 U, indicating α1-AAB positivity in these patients. With respect to the ETA-AAB, 14 (56%) patients were clearly positive (< − 8 U) and 3 (12%) patients exactly met the cut off with their ETA-AAB levels. Consequently, by the definition related to the cut off, 17 (68%) patients were positive for ETA-AAB, whereas 8 (32%) were negative for ETA-AAB. Because none of the patients that were negative for both GPCR-AAB, all 25 patients with prostate cancer were GPCR_AAB positive.

In contrast, ETA-AAB were undetectable in all (100%) and α1-AAB in 9 (90%) of the 10 controls. Interestingly, the one control with a strong increase in the α1-AAB level was the only one that presented with a defined additional diagnosis of psoriasis.

### Autoantibody characteristics

**Fig. 3:**
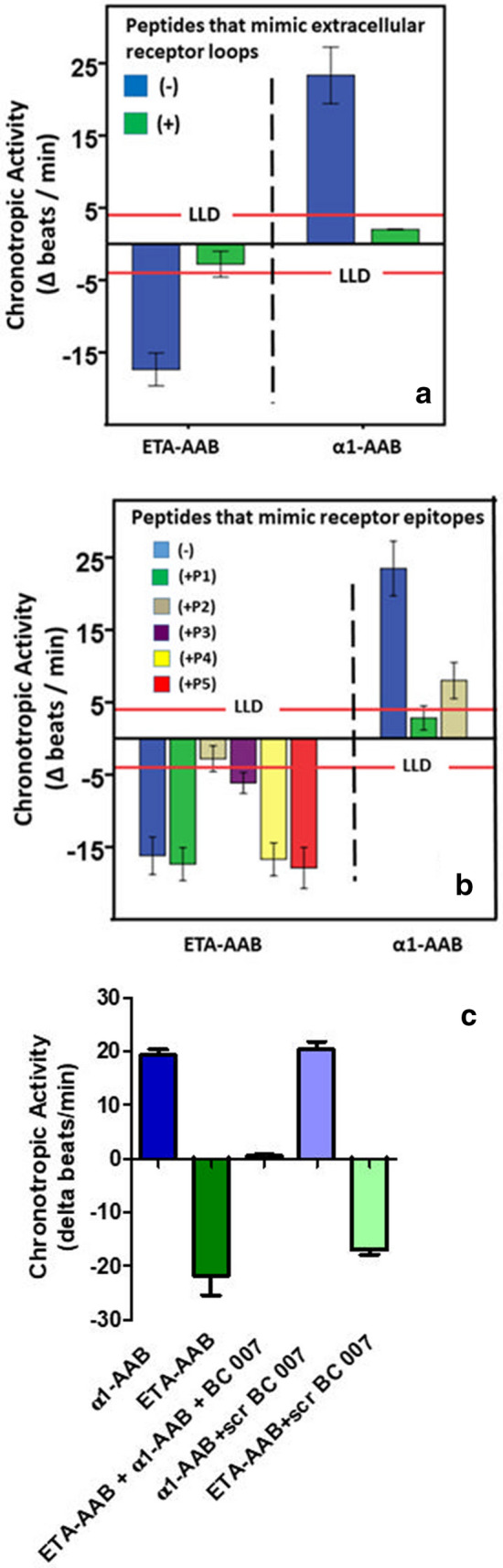
**a** Autoantibodies directed against the α1-adrenergic receptor (α1-AAB) and endothelin receptor A (ETA-AAB) of patients with prostate cancer target the first and second extracellular receptor loops, respectively, of their related receptors. Using the bioassay of spontaneously beating cultured neonatal rat cardiomyocytes, the chronotropic activity of the patients’ IgG (α1-AAB: n = 4; ETA-AAB: n = 5) was measured in the presence or absence of the competition of peptides representing the amino acid sequence of the extracellular receptor loops. Values below the lower limit of detection (LLD; α1-AAB = Δ 4 beats/min, ETA-AAB = Δ − 4 beats/min) were displayed as half range values. **b** Mapping of the first extracellular loop of the α1-adrenergic receptor and the second extracellular loop of the endothelin receptor A targeted by the related autoantibodies (α1-AAB, anti-α1-adrenergic receptor autoantibodies; ETA-AAB, anti-endothelin receptor A autoantibodies) of patients with prostate cancer. Using the bioassay of spontaneously beating cultured neonatal rat cardiomyocytes, the ETA-AAB activity and α1-AAB activity (mean ± SD) of the patients’ IgG (α1-AAB: n = 4; ETA-AAB: n = 5) was measured in the presence or absence of competing peptides that overlapped to represent the first extracellular loop of the α1-adrenergic receptor (P1: FWAFGR, P2: GRVFCDV) and the second extracellular loop of the endothelin receptor A (P1: FEYRGEQ, P2: EQHKTCM, P3: MLNATSK, P4: SKFMEFY, P5: FYQDVKD). Values below the lower limit of detection (LLD; α1-AAB = Δ 4 beats/min, ETA-AAB = Δ − 4 beats/min) were displayed as half range values. **c** *In vitro* neutralization by aptamer BC007 of autoantibodies directed against the α1-adrenergic receptor (α1-AAB) endothelin receptor A (ETA-AAB) of patients with prostate cancer. Using the bioassay of spontaneously beating cultured neonatal rat cardiomyocytes, the ETA-AAB and α1-AAB chronotropic activity of the patients’ IgG (n = 5) was measured in the presence or absence of the 0.1 µmol/L aptamer BC007 (sequence: GGTTGGTGTGGTTGG) or scrambled control (sequence: GGTGGTGGTTGTGGT). Values below the lower limit of detection (LLD; α1-AAB = ∆ 4 beats/min, ETA-AAB = Δ − 4 beats/min; cut off separating healthy subjects from patients: α1-AAB = Δ 8 beats/min, ETA-AAB = Δ − 8 beats/min) were displayed as half range values

As demonstrated in Fig. 3a for 4 (α1-AAB) and 5 (ETA-AAB), respectively, randomly selected patients, α1-AAB targeted the first extracellular receptor loop of α1-adrenergic receptor, and related to the blocking peptide, the subtype A; there specifically an epitope located tightly to the N-terminus of the receptor loop (Fig. 3b). For ETA-AAB, the second extracellular loop of their receptor was identified as the target (Fig. 3a) with an epitope located in the middle region of the loop. As shown in Fig. 3c for four patients that were positive for ETA-AAB and α1-AAB, pre-treatment of their IgG with the aptamer BC007 resulted in the loss of either α1-AAB and ETA-AAB.

## Discussion

The discovery of functionally active autoantibodies (GPCR-AAB) in the blood of patients that bind to GPCR for uncontrolled stimulation, which explains the perpetuation of pathogenically important downstream effects inside cells, has introduced a new class of autoimmune diseases denoted as functional autoantibody diseases. The bioassay of spontaneously beating cultured neonatal rat cardiomyocytes [23], is the most commonly used analytical tool for the identification and characterization of GPCR-AAB found in human serum.

Having recently shown with this bioassay that patients with BPH/LUTS carry functional autoantibodies, such as ETA-AAB, we used in the present study this tool to demonstrate that patients with prostate cancer also carry GPCR-AAB, but positive chronotropic α1-AAB in addition to the negative chronotropic ETA-AAB.

ETA-AAB were found in the majority (68%) of patients with prostate cancer, targeting the second extracellular receptor loop, an epitope located in the middle region of the loop. This is insignificantly different from our findings with a 60% ETA-AAB positivity in patients with BPH/LUTS in whom the ETA-AAB target was comparably localized [20].

In contrast to patients with BPH/LUTS, where ETA-AAB were the only GPCR-AAB, nearly all patients with prostate cancer additionally carried α1-AAB which targeted the first extracellular loop, and specifically an epitope localized near the loop’s N-terminus. α1-AABs were also frequently found in patients with idiopathic pulmonary hypertension, diabetes mellitus, drug-induced cancer [16, 17], psoriasis [25] and dementia [19], where the α1-AAB targeted the second extracellular receptor loop. However, α1-AAB targeting the first extracellular loop were found in dementia patients [26].

GPCR-AAB were not found in our control group, except one control who carried α1-AAB. Interestingly, this man suffered from psoriasis in which α1-AAB positivity was described [27].

Regarding the mechanisms to be considered for ETA-AAB- and α1-AAB-induced pathologies, many of these with the potency of generalization were discussed for ETA-AAB focused on systemic sclerosis [27] and for α1-AAB focused on Alzheimer’s disease [26].

For ETA-AAB in prostate cancer must be considered that their target, the ETA receptor, was identified in the prostate in the 1990s [28, 29], where its expression increased with tumor stage, grade and recurrence [30]. Overstimulation of the endothelin receptor A led to pathogenic effects involved in tumor growth, epithelial mesenchymal transition, apoptosis, metastasis, angiogenesis, and drug resistance, thus probably contributing to prostate carcinogenesis [2–5].

Unfortunately, a meta-analysis of clinical trials of ETA receptor antagonists for the treatment of hormone refractory prostate cancer did not show a significant benefit for overall or progression-free survival. However, patients benefited from a reduction in cancer-related bone pain and skeletal events [31].

To approximate the carcinogenic effects of α1-AAB, several arguments for the effects of α1-adrenergic receptor-mediated signaling on the development, progression and prevention of prostate cancer were summarized in [7].

The α1-adrenergic receptor subtype A is localized in the prostate [32, 33] and mRNA and receptor increase were found in the aging gland [34, 35]. Proliferation in prostate cancer epithelium was demonstrated after α1-adrenergic receptor stimulation [36]. Due to potentially carcinogenic effects seen after stimulation of the α1-adrenergic receptor in prostate cell lines, suitable blockers have been proposed for the treatment of prostate cancer [37]. Retrospective cohort and observational studies did indeed show a reduced incidence of prostate cancer if patients treated with α1- adrenergic receptor blockers for hypertension and/or benign prostate hyperplasia; in the latter patients the blockers increased the apoptotic index and reduced the vascularity of the prostate tumor [36].

For some antitumor effects of the α1-adrenergic receptor antagonists, however, in addition to the patient benefit discussed as a result of the α1-adrenergic receptor antagonization, a DNA breaking activity of the blockers, which leads to mitotic arrest of the cell cycle and mitochondrial damage, must be considered [37].

By combining ETA-AAB and α1-AAB in prostate cancer with the downstream effects after agonist binding and the lack of prevention of GPCR over-stimulation after GPCR-AAB binding [15], we postulate the establishment of GPCR-AAB/GRCR axes that should be more potent than the physiological axes in over-stimulation and induction of pathologies.

**Fig. 4:**
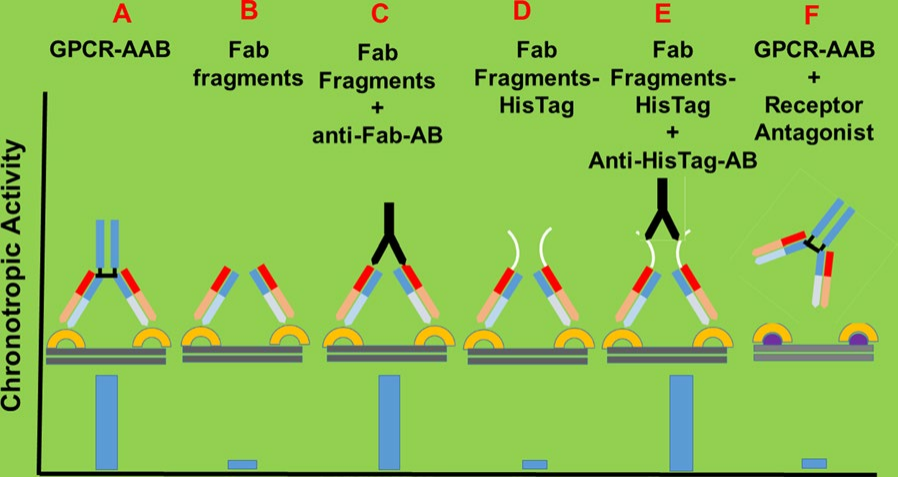
Cross-linking of G-protein coupled receptors (GPCR) by the corresponding functional autoantibodies (GPCR-AAB); the key event for receptor mediated signaling after receptor binding of GPCR-AAB is illustrated [21] Using the bioassay of spontaneously beating cultured neonatal rat cardiomyocytes, the chronotropic activity of GPCR-AAB in the absence (**a**) and presence of a related receptor blocker (**f**) as well as of monomeric Fab fragments (**b**), monomeric Fab fragments cross-linked by anti-Fab-antibodies (**c**), monomeric Fab fragments coupled to polyhistidine tag (**d**) and Fab fragments coupled to polyhistidine tag cross-linked by polyhistidine antibodies

As illustrated in Fig. 4 (using data published in [38–40]), the different binding sites were considered the reason for the discrepancy between physiological agonists and GPCR-AAB in GPCR stimulation and control. While physiological agonists bind in a hydrophobic pocket of GPCR, GPCR-AAB binds to the extracellular loops and, due to the bivalent nature of IgG, crosslink the GPCR to realize the functional activity of GPCR-AAB without control.

It remains speculative, however, whether the postulated creation of the α1-AAB/α1-adrenergic and ETA-AAB/ETA receptor axes in prostate cancer patients overpowers the drug-induced receptor antagonization that was intended to benefit patients.

However, it should not be concealed that, derived mainly from studies of cardiovascular diseases associated with GPCR-AAB, there were convincing data that demonstrated a more pronounced patient benefit if antagonist treatment was combined with an attack on the GPCR-AAB [41].

In case future studies manifest the pathogenic roles of α1-AAB and ETA-AAB in prostate cancer, a treatment strategy targeting the GPCR-AAB should be considered. As a first step of such treatment strategy, we demonstrated the in vitro neutralization of the prostate cancer associated GPCR-AAB with the drug BC 007.

The recently successfully completed phase 1 clinical trial with BC 007 in combination with the successful demonstration of GPCR-AAB neutralization in humans [42, 43] could open the door for studies to evaluate in vivo GPCR-AAB neutralization with BC 007 as a new complementary therapeutic strategy for patients with prostate cancer.

## Conclusion

Of patients with prostate cancer, nearly 96% of them carried positive chronotropic autoantibodies directed against the first extracellular loop of the α1-adrenergic receptor (α1-AAB) and 68% negative chronotropic autoantibodies directed against the second extracellular loop of the endothelin receptor A (ETA-AAB). We postulate α1-AAB/α1-adrenergic receptor and ETA-AAB/endothelin receptor A axes for these patients, that, in contrast to the related axes based on the physiological receptor ligands, lack control mechanisms, such as receptor desensitization and downregulation to counteract over-boarding stimulation.

Therefore, the stimulation of both GPCR-AAB axes in patients with prostate cancer should long-lasting activate downstream pathways whose carcinogenic potency is probably more potent as compared to the axes considering physiological ligands.

Last but not least, we believe that treatment strategies that counteract α1-AAB and ETA-AAB could complement the standard treatment for patients with prostate cancer. In this context, the in vivo neutralization of α1-AAB and ETA-AAB by treatment with the aptamer BC 007 could be an option for the future.

## Data Availability

All data generated or analyzed during this study are included in this published article.
